# Retracted: Designing a Bioengine for Detection and Analysis of Base String on an Affected Sequence in High-Concentration Regions

**DOI:** 10.1155/2021/3845370

**Published:** 2021-02-26

**Authors:** BioMed Research International

*BioMed Research International* has retracted the article titled “Designing a Bioengine for Detection and Analysis of Base String on an Affected Sequence in High-Concentration Regions,” [1], as it was found to be a copy, without referencing, of the article: Syed Mahamud Hossein and A K Bandyopadhyay, “Bio-Software Engine Analysis for High Concentration Regions (HCR) Detection of a Fixed Base String on an Affected Sequence” Int. J. of Applied Information Systems, 2013, Vol. 5, No. 1, pp. 61-68. doi: 10.5120/ijais12-450726 [2].

The first author approached us and offered to rephrase and update their article [1], however the editorial board advised that the article should be retracted. The second author says the submission was his responsibility and is his work.
